# Rat Bite Fever: It’s Still a Threat

**DOI:** 10.1016/j.jhsg.2025.100901

**Published:** 2026-01-02

**Authors:** Alexander J. Bergeson, Kyler A. Hardie, Robert E. Van Demark, Matthew C. Anderson

**Affiliations:** ∗ Sanford School of Medicine (SSOM), University of South Dakota, Vermillion, SD; † University of North Dakota, Grand Forks, ND

**Keywords:** Haverhill fever, Rat bite, Sodoku, Spirillum minus, *Streptobacillus moniliformis*

## Abstract

*Streptobacillus moniliformis* is considered normal flora of the upper respiratory tract of rodents and is the predominant cause of rat bite fever in North America. Although patients with rat bite fever oftentimes present with migratory polyarthralgia, cultures of affected joints do not grow bacteria in the vast majority of cases. We present a case of a 32-year-old woman who initially presented with a painful, swollen right index finger proximal interphalangeal (PIP) joint following a bite from her pet rat. After initial treatment with empiric antibiotics did not clear the infection, PIP joint irrigation and debridement revealed culture-positive septic arthritis. This case illustrates the patient’s unusual presentation of *S*. *moniliformis* infection through septic arthritis of the PIP joint.

Rat bite fever is a febrile, systemic disease that typically presents with a classic triad of relapsing fevers, rashes, and migratory polyarthralgia.^1^, ^2^, ^3^, ^4^, ^5^ Typically occurring following a bite from a rodent, two different bacteria cause rat bite fever. Of those bacteria, *Streptobacillus moniliformis* is the predominant cause in North America, and *Spirillum minus* is the main cause of disease in Asia. Both causative organisms are considered normal flora of the rodent’s upper respiratory tract.^1^^,^^2^ Although rat bite fever commonly causes joint pain presenting as migratory polyarthralgia, rarely do cultures of the affected joints grow bacteria.^6^^,^^7^ We report a case of culture-positive *S*. *moniliformis* septic arthritis in a patient with a recent history of a rat bite to the index finger.

## Case Report

**Figure 1:**
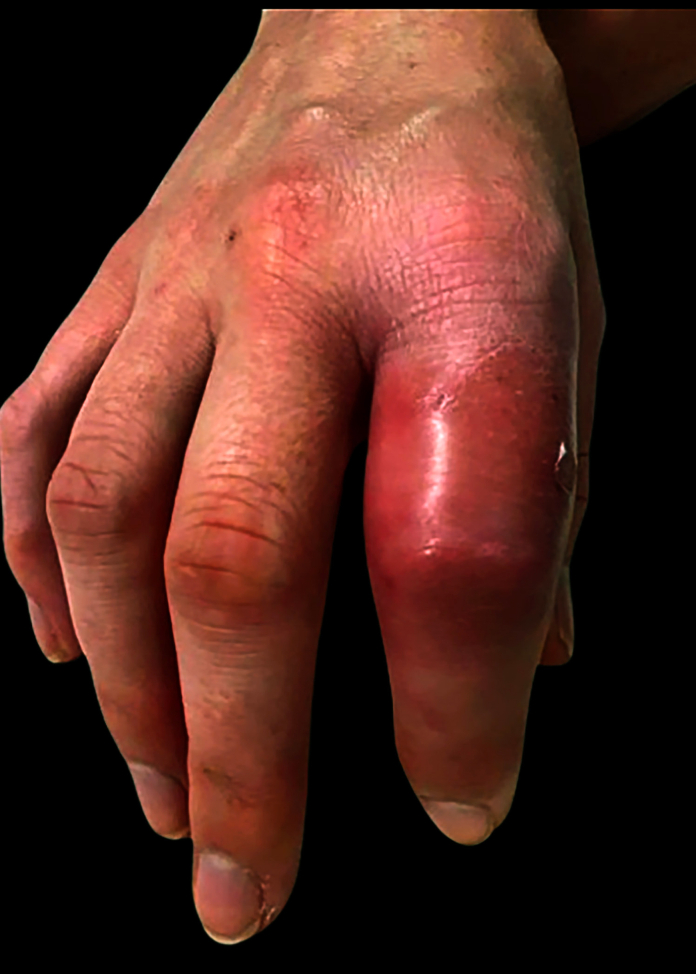
Initial patient presentation 8 days following a rat bite to the right index finger.

A 32-year-old woman presented to an acute care clinic with a painful, swollen right index finger following a bite to the area 8 days prior by her pet rat. Along with pain and swelling, the patient had a fever, chills, body aches, and a white blood cell count of 11,800. Radiographs showed only soft tissue swelling of the finger. Following the visit, the patient received a prescription of doxycycline for suspected cellulitis and a referral to the orthopedic hand clinic. Presenting at the hand clinic 3 days later, the patient had two scabs from the rat bite located at the mid-level of the proximal phalanx, along with redness and swelling of the proximal interphalangeal (PIP) joint (Fig. 1). Irrigation and debridement was recommended and performed later that day. Frank pus was present in the PIP joint. Culture swabs collected from the PIP joint grew gram-negative *bacilli*, later confirmed to be *S*. *moniliformis*. The patient was then admitted to the hospital for observation, receiving 2 g of intravenous (IV) Rocephin daily.

**Figure 2:**
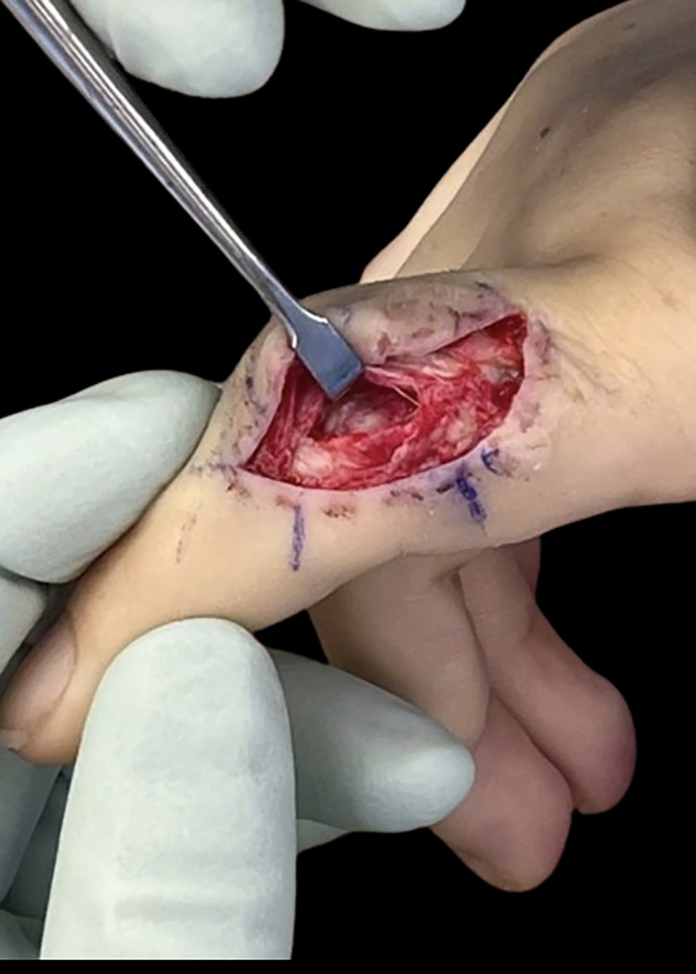
Intraoperative photograph of right index finger.

**Figure 3:**
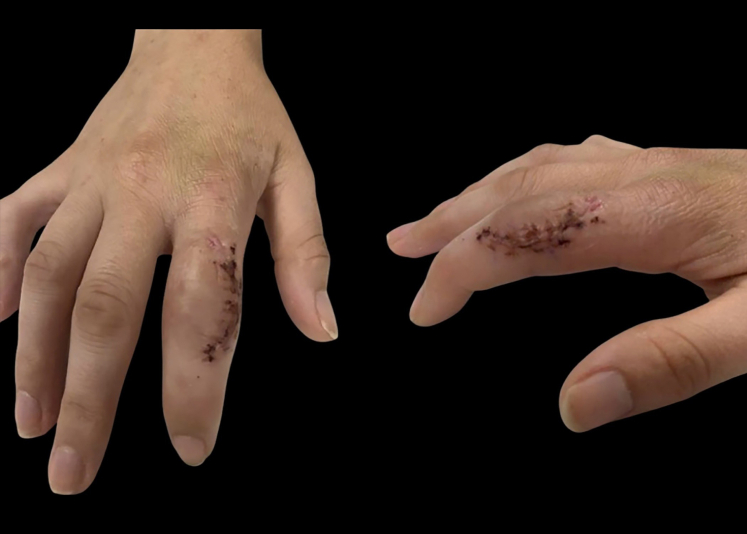
Clinical photographs from the 2-week post-op visit.

Two days after the initial surgery, a second irrigation and debridement of the right index finger was performed (Fig. 2). Cultures from the second surgery showed no growth. The patient was discharged from the hospital and continued outpatient IV Rocephin for 10 days. After completing the initial course of outpatient antibiotics, the patient followed up with both infectious disease and orthopedics. At the orthopedic follow-up 2 weeks post-op, the patient reported decreased pain and swelling in the finger (Fig. 3). Active PIP range of motion at that time measured from 20° to 43° of flexion. The patient began occupational therapy and was fitted for a nighttime extension splint. During infectious disease follow-up, IV Rocephin was switched to oral amoxicillin, which was continued for 3 weeks.

**Figure 4:**
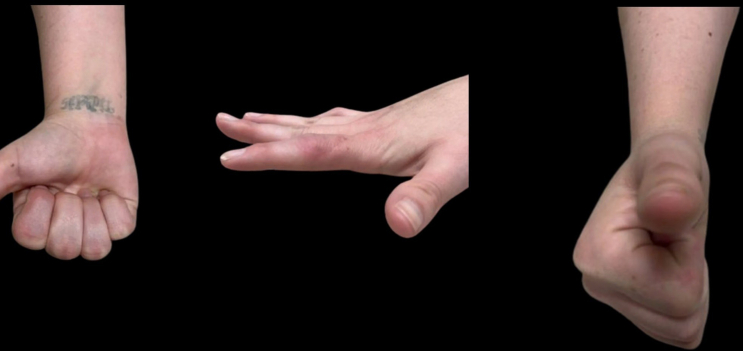
Clinical photographs from the 3-month postoperative clinic visit.

At her 3-month follow-up examination, there was minimal swelling of the finger, and the PIP joint range of motion was from 10° to 90° of flexion (Fig. 4).

## Discussion

*S*. *moniliformis* is a nonmotile, filamentous, gram-negative, highly pleomorphic, non–acid-fast rod.^1^, ^2^, ^3^ Commonly found in the respiratory tracts of mice and rats, *S. moniliformis* spreads to humans through rodent bites or contact with rodent saliva.^1^, ^2^, ^3^ Following spreading to humans, *S*. *moniliformis* typically presents through three main infectious disease processes.

The two most common diseases associated with *S. moniliformis* include rat bite fever and Haverhill fever. Both diseases present with a similar set of symptoms but vary in the transmission of disease. Rat bite fever, the more common of the two diseases, presents following a bite from a rodent, typically a rat or mouse.^1^, ^2^, ^3^ Through the bite, *S. moniliformis* from the rodent’s respiratory tract can then enter the bloodstream and cause systemic disease.^1^, ^2^, ^3^ Haverhill fever, on the other hand, is contracted through consuming food or drink contaminated with *S. moniliformis*.^1^, ^2^, ^3^ Although rat bite fever and Haverhill fever have different modes of transmission, they both present with a similar set of symptoms. Following an incubation period ranging from 3 to 10 days, both diseases typically present with a triad of fever, rash, and migratory polyarthralgia.^1^, ^2^, ^3^, ^4^, ^5^ Other signs of infection may include headache, sore throat, vomiting, and the most serious complication of endocarditis.^2^, ^3^, ^4^ If left untreated, the mortality rate can approach 13%,^1^^,^^2^ making it important to recognize and treat promptly. Despite *S. moniliformis* infections being potentially life-threatening if untreated, the total yearly number of cases in the United States is widely unknown. Kache et al^4^ estimated 0.20 cases of rat bite fever per 1,000,000 individuals in the United States between 2001 and 2015. The authors also acknowledged that the true number of cases is likely underestimated due to the vague presentation of the disease and because rat bite fever is not a reportable disease in the United States.^4^

Less frequently, *S. moniliformis* can cause septic arthritis, as seen in this case presentation. Previous research has proposed that septic arthritis and rat bite fever function as two entirely different forms of infection from *S. moniliformis*.^7^^,^^8^ During rat bite fever, joint pain typically presents as migratory polyarthralgia.^1^, ^2^, ^3^, ^4^, ^5^ Under close examination, migratory polyarthralgia presents in a similar fashion as rheumatoid arthritis with the presence of synovial hyperplasia, inflammatory cell infiltration, and pannus formation.^3^ Additionally, positive joint cultures during rat bite fever are extremely rare.^6^, ^7^, ^8^ Because of those two findings, migratory polyarthralgia during rat bite fever is suspected to be immune mediated.^8^ Septic arthritis, on the other hand, is believed to be derived from direct infection of the joint space, thereby causing pyogenic arthritis.^7^^,^^8^ Septic arthritis with positive cultures of *S. moniliformis* from the joint space is very rare. Dendle et al^7^ found 15 reported cases worldwide of *S. moniliformis* septic arthritis from 1985 to 2006. Of those cases, the majority presented in cases of polyarticular septic arthritis. Monoarticular septic arthritis, similar to this case, is much less common.^6^^,^^7^ Although the lack of positive cultures during rat bite fever is believed to be due to the lack of true infection of the joint, inadequate culture technique or poor joint aspiration for culture could also represent why *S. moniliformis* is rarely cultured from synovial fluid.^6^^,^^7^

Current recommendations for treatment of *S. moniliformis* infections suggest Penicillin G as the first-line treatment.^1^^,^^2^^,^^8^ Despite that, *S. moniliformis* is typically sensitive to a wide variety of antibiotics.^5^ Other commonly used treatment regimens include doxycycline^2^ and ceftriaxone,^3^^,^^5^ both of which were utilized in this case. Ultimately, the treatment of *S. moniliformis* infections does not rely heavily on antibiotic choice. Instead, it relies on early recognition of vague clinical signs and clinical history of exposure to rodents to recognize the presence of the infection and the subsequent need for antibiotic treatment.^1^

This case highlights a rare case of monoarticular septic arthritis due to *S. moniliformis.* In cases of suspected infection from *S. moniliformis*, prompt empiric antibiotic treatment should be initiated to prevent severe progression of infection. In rare cases where a bite lies near a joint, as in this case, clinicians should consider the possibility of septic arthritis, in which irrigation and debridement may be necessary to treat the infection. In both cases, failure to recognize and treat the infection may lead to the increased patient morbidity and potentially patient mortality.

Written consent has been obtained from the patient for publication.

## Conflicts of Interest

No benefits in any form have been received or will be received related directly to this article.
